# Complete Genome Sequence of a Novel Genotype of *Pepper Mild Mottle Virus* Infecting Pepper in Chile

**DOI:** 10.1128/MRA.01183-20

**Published:** 2020-11-19

**Authors:** Sven M. H. Berendsen, Willem E. W. Schravesande

**Affiliations:** a Rijk Zwaan Breeding B.V., De Lier, the Netherlands; DOE Joint Genome Institute

## Abstract

The complete genome sequence of a *Pepper mild mottle virus* (PMMoV) found on pepper seeds produced in Chile in 2019 was determined using Oxford Nanopore Technologies and Sanger sequencing. The low nucleotide sequence identities (between 89% and 91%) to known PMMoV isolates suggested that this isolate belongs to a novel genotype.

## Abstract (precis)

The complete genome sequence of a *Pepper mild mottle virus* (PMMoV) found on pepper seeds produced in Chile 2019 was determined using Oxford Nanopore Technology and Sanger sequencing. The low nucleotide sequence identities (between 89% and 91%) to known PMMoV isolates suggested that this isolate belongs to a novel genotype.

## ANNOUNCEMENT

*Pepper mild mottle virus* (PMMoV), of the genus *Tobamovirus* in the family *Virgaviridae*, is a seed-borne virus infecting pepper crops (*Capsicum* spp.) worldwide (1). PMMoV pathotypes can be categorized into three subgroups, P_1,2_, P_1,2,3_, and P_1,2,3,4_, based on the isolate response to the corresponding resistance genes controlled by L^1^, L^3^, and L^4^ (2–4). In May 2019, a seed quality assessment was conducted for the presence of tobamoviruses on a pepper seed lot produced and harvested in April 2019 in the Valparaíso Region of Chile. Using the method described by ISHI-Veg 2019 (5), a *Tobamovirus* (PRO54348) was detected by local lesion formation on the leaves of Nicotiana tabacum L. cv. Xanthi NN. Viral RNA was extracted from the local lesions using a Qiagen RNeasy plant minikit. Using reverse transcription-PCR (RT-PCR) targeting the coding DNA sequence (CDS) of the coat protein (6, 7) and sequencing the amplicons using the Sanger technique showed a maximum identity of 91% with known PMMoV isolates. The pathotype of isolate PRO54348 was determined by mechanically inoculating pepper varieties with different L resistance genes, which identified it as a PMMoV-P_1,2_. This suggested that there is more genetic diversity among PMMoV-P_1,2_ isolates than currently known. To obtain the full-genome sequence from isolate PRO54348, the PMMoV systemic host Nicotiana benthamiana was inoculated with the positive seed lot using the same ISHI-Veg 2019 method. After 1 week, viral RNA was extracted from the symptomatic N. benthamiana leaf material using a Qiagen RNeasy plant minikit. The RNA was used as the template for double-stranded cDNA synthesis using a Maxima H Minus double-stranded (ds) cDNA kit (Thermo Scientific). A cDNA library was constructed following the manufacturer’s protocol (LSK-109, Oxford Nanopore Technologies) and sequenced on R9.4 MinION flow cells for 72 h. After 72 h of sequencing, the data were base called using Guppy v. 3.6 (Oxford Nanopore Technologies) and trimmed/filtered based on length and quality scores (q = 6; minlength = 1000; headcrop = 20; tailcrop = 20) using Porechop v. 0.2.4 (https://github.com/rrwick/porechop) and Nanopolish v. 0.13.2 (https://github.com/jts/nanopolish). The average read length was 2,847 bp, and the total number of reads was 3,116,876. All tools were run with default parameters unless otherwise specified. The filtered base-called reads were assembled using CLC Genomics Workbench v. 12.0 (Qiagen) using the *de novo* assembly option in the long-read module (beta). No polishing steps were executed prior to assembly. A mean coverage of 4,587× was obtained. The assembled genomic sequence was confirmed and polished through Sanger sequencing of additional RT-PCR amplicons along the genomic RNA. The 5′ and 3′ termini of the viral RNA were obtained using rapid amplification of cDNA ends (RACE) technology. The 5′ terminal end was amplified following the manufacturer’s protocol using the 5′/3′ RACE kit, 2nd generation (Roche). For the amplification of the 3′ terminal, the molecules were tailed with dATP and terminal transferase (TdT). These tailed molecules were used as input material for 3′ RACE using the manufacturer’s protocol. The complete genome sequence for isolate PRO54348 was obtained and further analyzed and annotated by using a PMMoV reference genome (GenBank accession number NC_003630) as the reference sequence in the Whole Genome Alignment (beta) toolbox using default settings in CLC Genomics Workbench v. 12.0. The genome comprised 6,356 nucleotides with a GC content of 41.6%. Using the complete genome sequence, PRO54348 was found to share only 89 to 91% sequence identity with all known PMMoV isolates available on NCBI using BLASTN. The genome encodes four proteins, a 183-kDa protein of 1,612 amino acids (aa), a 126-kDa protein of 1,117 aa, a movement protein of 257 aa, and a coat protein of 157 aa. These four proteins share up to 97%, 97%, 92%, and 96% sequence identity, respectively, with all known PMMoV isolates available on NCBI using BLASTP. The low level of sequence identity on the RNA level but higher sequence identities for the four proteins suggest that PRO54348 should be considered a novel genotype of PMMoV.

### Data availability.

The complete genome sequence of PRO54348 has been deposited in GenBank under the accession number MT385868. The raw reads were deposited under BioProject accession number PRJNA669436, run number SRR12834856.
